# Four types of scrapie in goats differentiated from each other and bovine spongiform encephalopathy by biochemical methods

**DOI:** 10.1186/s13567-019-0718-z

**Published:** 2019-11-25

**Authors:** Jan P. M. Langeveld, Laura Pirisinu, Jorg G. Jacobs, Maria Mazza, Isabelle Lantier, Stéphanie Simon, Olivier Andréoletti, Cristina Acin, Elena Esposito, Christine Fast, Martin Groschup, Wilfred Goldmann, John Spiropoulos, Theodoros Sklaviadis, Frederic Lantier, Loukia Ekateriniadou, Penelope Papasavva-Stylianou, Lucien J. M. van Keulen, Pier-Luigi Acutis, Umberto Agrimi, Alex Bossers, Romolo Nonno

**Affiliations:** 1 Wageningen BioVeterinary Research (WBVR), Wageningen University & Research, Houtribweg 39, 8221RA Lelystad, The Netherlands; 2 Department of Veterinary Public Health and Food Safety, Istituto Superiore di Sanita (ISS), 299-00161 Rome, Italy; 3 Italian Reference Centre for TSEs, Istituto Zooprofilattico Sperimentale del Piemonte (IZSTO), 10154 Turin, TO Italy; 4 UMR 1282 ISP, Institut National de la Recherche Agronomique (INRA), University of Tours, 37380 Nouzilly, France; 5 Commissariat à l’Énergie Atomique (CEA), 91191 Gif-sur-Yvette, France; 6 UMR INRA/ENVT 1225 IHAP, École Nationale Vétérinaire de Toulouse (ENVT), 31300 Toulouse, France; 7 Research Centre for TSE and Emerging Transmissible Diseases, University of Zaragoza (UNIZAR), 50013 Zaragoza, Spain; 8 Friedrich-Loeffler-Institut (FLI), Institute of Novel and Emerging Infectious Diseases, Greifswald-Isle of Riems, 17493 Greifswald, Germany; 9 The Roslin Institute and Royal (Dick) School of Veterinary Studies, University of Edinburgh (UEDIN), Easter Bush, Midlothian, EH25 9RG UK; 10 Department of Pathology, Animal and Plant Health Agency (APHA), Woodham Lane, Addlestone, Surrey KT15 3NB UK; 11 School of Pharmacy, Aristotle University of Thessaloniki (AUTh), 54124 Thessaloniki, Greece; 12 Hellenic Agricultural Organization DEMETER, Veterinary Research Institute, 57001 Thessaloniki, Greece; 13 Veterinary Services (VSC), Ministry of Agriculture, Rural Development and Environment, 1417 Nicosia, Cyprus

## Abstract

Scrapie in goats has been known since 1942, the archetype of prion diseases in which only prion protein (PrP) in misfolded state (PrP^Sc^) acts as infectious agent with fatal consequence. Emergence of bovine spongiform encephalopathy (BSE) with its zoonotic behaviour and detection in goats enhanced fears that its source was located in small ruminants. However, in goats knowledge on prion strain typing is limited. A European-wide study is presented concerning the biochemical phenotypes of the protease resistant fraction of PrP^Sc^ (PrP^res^) in over thirty brain isolates from transmissible spongiform encephalopathy (TSE) affected goats collected in seven countries. Three different scrapie forms were found: classical scrapie (CS), Nor98/atypical scrapie and one case of CH1641 scrapie. In addition, CS was found in two variants—CS-1 and CS-2 (mainly Italy)—which differed in proteolytic resistance of the PrP^res^ *N*-*terminus*. Suitable PrP^res^ markers for discriminating CH1641 from BSE (C-type) appeared to be glycoprofile pattern, presence of two triplets instead of one, and structural (in)stability of its core amino acid region. None of the samples exhibited BSE like features. BSE and these four scrapie types, of which CS-2 is new, can be recognized in goats with combinations of a set of nine biochemical parameters.

## Introduction

Prion diseases or transmissible spongiform encephalopathies (TSEs) are lethal neurological infections in mammals caused by prions from either sporadic, familial or transmissible origin [1, 2]. Since the 1980s, a zoonotic form of the disease emerged in cattle as bovine spongiform encephalopathy (BSE, C-type) through consumption of contaminated meat and bone meal (MBM) [3, 4]. BSE was detected in the United Kingdom but later also in and outside of Europe although less frequently. In 1995, a human variant form of human Creutzfeldt-Jakob disease (vCJD) emerged with phenotypic similarities to BSE [5, 6]. A decennium later, TSE in cattle was differentiated by Western blotting in three types of BSE, C-type BSE and rare cases of H- and L-type BSE [7–9]. Measures to prevent continual feeding of livestock with MBM circulation have led to the near disappearance of BSE and vCJD worldwide. Critical herein were also diagnostic post mortem tests with prion protein (PrP) specific antibodies that reveal the presence of protease resistant prion material that is composed of malformed PrP (PrP^Sc^) [10]. Awareness and strict surveillance of prion infections remain necessary, not only because of the zoonotic and epizootic risks of BSE but also other forms of TSE with different transmittabilities such as chronic wasting disease (CWD) in cervids in North America and South Korea, and newly discovered TSEs in cervids in Norway and camelids in Algeria [11–13].

Like other infectious agents, prions also exist as strains. Their transmissibility depends uniquely and largely on the amino acid sequence of normal cellular PrP (PrP^C^), and possibly on host factors during conversion of PrP^C^ to PrP^Sc^ [14]. Strain characteristics are phenotypical properties such as incubation time, lesion profile, and variations in deposition and molecular features of PrP^Sc^. Multiple strains from scrapie in sheep have been described in rodent bioassays, while bovine BSE behaves as a single strain [1, 5, 15].

However, in goats strain typing efforts have rarely been reported [16–18]. While scrapie in sheep is known to have existed for centuries, there are no indications that under natural conditions other species are infected by scrapie except goats. The source of the BSE epidemic is still uncertain, but plausible explanations are that it has evolved from small ruminant scrapie or from a sporadic case of BSE in cattle [3, 9]. Sheep and goats are known to be susceptible to BSE, but in the field only two cases in goats have been reported and these most probably originated from ingesting BSE contaminated feed [19–21].

Before deciding to carry out strain typing bioassays in rodents with their long lasting incubation times, ELISA and Western blotting (WB) with infected brain samples are important to rapidly classify scrapie like TSE types^1^ and to exclude the presence of BSE [22–30]. In sheep, scrapie occurs in different biochemical types such as classical scrapie^2^ (CS), atypical/Nor98 scrapie (AS) and a rare form of CS, CH1641 scrapie. Proteolytic digestion with proteinase K (PK) of the PrP^Sc^ aggregate and its subsequent unfolding and dissociation are essential for binding by PrP site-specific antibodies. CH1641 scrapie exhibits similarities with BSE since in both types distinct *N*-*terminal* PrP epitopes are protease sensitive [31]. In addition, mixtures of TSE forms could be present in a single animal, which hamper recognition of low BSE levels [32].

During 2004–2014, we collected over seventy TSE goat brain samples from seven European countries based on various criteria such as tissue quality, geographical distribution, breed, *PRNP* genotype. From this unique collection, over thirty goat TSE isolates from seven EU countries have been subjected to biochemical TSE-typing. These samples were probed by ELISA and Western blotting for the presence of different sequence domains in PrP^Sc^ under different conditions of pre-treatment and proteolysis when preparing its proteinase K (PK) resistant domain (PrP^res^). Samples such as CS, AS, BSE and CH1641 scrapie served as references. These materials are also under strain typing investigation by rodent bioassays.

## Materials and methods

### Antibodies

PrP-specific monoclonal antibodies (mAbs) used in this study were L42 and P4 (R-Biopharm, Germany), Sha31, SAF84, SAF34 and Bar224 (SpiBio, France), and 12B2 and 9A2 (WBVR, Lelystad, Netherlands). The mapped epitope amino acid sequences (sheep PrP numbering, [33]) determined by immobilized multi-peptide analyses are: 70QPHGGGW76 (SAF34), 93WGQGGSH99 (P4), 93WGQGG97 (12B2), 102WNK104 (9A2), 144FGSNDYEDRYYR154 (Bar224), 148YEDRYY153 (L42), 148YEDRYYRE155 (Sha31), and 167YRPVDQY172 (SAF84) [34–38].

### Animals and tissues

During 2004–2012, we collected over seventy TSE goat brain samples from seven European countries fitting the EU rules EC No. 999/2001 for TSE surveillance. As study samples a selection of 32 of these field cases was chosen together with two confirmed negatives (study codes G15, G17), and three experimentally infected goats: orally challenged with goat scrapie (F11), goat intra-cerebrally (i.c.) inoculated with sheep scrapie (F2) and i.c. inoculated with bovine BSE (ic-gtBSE1) (Table 1). The selection was based on criteria such as tissue quality, genotype, broad geographical distribution, and potential type variation. Tissues used consisted mainly of brain stem obtained at slaughterhouses or at euthanasia of experimentally infected animals. The national identity code, country of origin, breed, age and PrP genotype of the samples were recorded. Only the samples from United Kingdom, Netherlands, and two Greek cases (G13, G16) originated from single holdings.

**Table 1:** **Goat sample codes and details and final outcome of the TSE typing study**

Study code	Country	Identity #	Breed [region]^a^	Age (year)^a^	Genotype^b^	Molecular TSE-type
STUDY CASES						
I2	IT	114921/1/1	Camosciata [Piedmond]	10	240PP	CS-2
I3		121429/1/1	Meticcia [Sicily]	5	240PP	CS-2
I4		128710/1/1	Saanen [Lombardy]	3	211QR, 240PS	CS-2
I5		17646/1/1	Meticcia [Sicily]	5	240PP	CS-2
I7		85788/1/1	Meticcia [Sicily]	> 1.5	240PP	CS-2
I9		85792/1/1	Meticcia [Sicily]	6	143HR, 240PS	CS-2
I11		117463/1/1	Meticcia [Emilia-Romagna]	9	240PS	CS-2
I12		144508/1/1	Alpina [Apulia]	5	240PS	CS-2
I15		87016/1/1	Meticcia [Campania]	6	154RH, 240PS	AS
N1	NL	577277	Dwarf goat [Limburg]	2–3	143HR, 240PS	CS-1
N2		586632-32	Dwarf goat [Limburg]	?	240PP	CS-1
N3		586632-33	Dwarf goat [Limburg]	?	143HR, 240PS	CS-1
F2*	FR	CP40	Saanen [INRA]	4	240PS	CS-1
F3		CDP1028	Saanen [Poitou]	5	240PP	CS-1
F6		CP2119	Saanen [Charentes]	3–4	240PS	CS-1
F10		CP/2143	Alpine [Limousin]	3–4	240PS	CS-1
F11*		CP2154	Saanen [Poitou]	4	142IM, 240PP	CS-1
F14		CP9041	Alpine [Poitou]	6	142IM, 240PS	CS-1^c^
F16		CP9135	Alpine [Indre]	6	240PS	CS-2
ic-gtBSE1*		CH1075	Saanen [INRA]	?	211RQ, 240PS	BSE
S2	SP	C-163P	Alpine [Asturia]	6	240PS	CS-1
S3		C-645P	Crossbreed [Aragon]	4	240PP	CS-1
G2	GR	1663	*Capra prisca* [Macedonia]	?	240PP	CS-1^c^
G3		1676	*Capra prisca* [Macedonia]	4	143HR, 240PP	CS-1
G11		GR005	*Capra prisca* [Larissa]	6	211RQ, 222QK	CS-1
G12		GR177	*Capra prisca* [Larissa]	4	222QK	<^c^
G13		GR018	*Capra prisca* [Larissa]	5	wt	CS-1
G14		GR055	*Capra prisca* [Ioannina]	4	wt	CS-1
G15		GR195	*Capra prisca* [Kozani]	2.5	222QK	NEG
G16		GR091	*Capra prisca* [Thessaloniki]	2	wt	CS-1
G17		GR247	*Capra prisca* [Evros]	4.6	222QK	NEG
C1	CYP	Zyp13	Damascus [Nicosia]	4	240PP	CS-1
C2		Zyp21	Damascus [Nicosia]	5	240PP	CS-1
C3		Zyp27	Damascus [Nicosia]	3	wt, 240PP	CS-1
UK-A2	UK	G08-1475	Anglo-NubianxSaanen [?]	4	127GS, 240PP	CS-1
UK-B2		G08-1469	Anglo-NubianxSaanen [?]	8	127GS, 240PP	CS-CH1641
UK-C2		G08-1460	Anglo-NubianxSaanen [?]	9	127GS, 240PP	CS-1
UK-D2		G08-1446	Anglo-NubianxSaanen [?]	7	211RQ, 240PP	<^c^

Study codes will be used in the text reflecting the country of origin, I for Italy, N Netherlands, F France, S Spain, G Greece, C Cyprus, UK United Kingdom. From single holdings were only the cases from Netherlands, UK and Greek cases G13 and G16. Symbols: *, obtained after experimental infections (see “Materials and methods”). The specific tests performed on the samples, the PrP^res^ content of the sample (when analysed in Triplex-WB) are presented in Additional file 2.

^a^INRA, Institut National de la Recherche Agronomique. ? = region of origin or age not disclosed or not known.

^b^Genotype as defined by specific polymorphic codon positions in the goat *PRNP* gene. Wild type (wt) is defined as follows: 127GG, 142II, 143RR, 154RR, 211RR, 222QQ, 240SS. Unless wt genotype, only the codon positions that differ from homogenous wild type are shown.

^c^Samples F14 and G2: probably CS-1. F14 too weak for Triplex-WB and ISS-PK methods, G2 too little amount for distribution. G12 and UK-D2: late samples, only analysed by Triplex-WB, too weak signal for analysis.

In addition, infected goat brain materials from other studies were investigated derived from animals infected i.c. with scrapie (*n* = 6) and orally or i.c. with BSE (*n* = 9) [39, 40]. As occasional reference controls were included experimental sheep and goat BSE, ovine CS (*n* = 2), bovine BSE, caprine AS, and i.c. raised CH1641 material from sheep (*n* = 2) and goat, and an ovine CH1641-like field case (see Additional file 1). The animal experiments to obtain these materials were performed at WBVR according to European directive 2010/63/EU and in agreement with the Dutch Central Authority for Scientific Procedures on Animals, permit number AVD401002016522.

Fifty percent macerates in water were prepared under TSE sterile conditions. Samples were weighed, immersed in an equal part of water, minced, and left for 18 h at 4 °C. Material was ground in a Pyrex glass Dounce to a homogenous paste, further forced several times through a 19G needle, and finally stored in aliquots at −80 °C. These macerates were dispatched to the participating laboratories. Depending on the analysis and the timely availability of tissue, the set of samples used per study differed (see Additional file 2).

### CEA-ELISA (performed at CEA: Commissariat à l’énergie atomique et aux énergies alternatives)

A discriminatory ELISA for detecting BSE in small ruminants followed procedures as described [28]. Each sample was treated in two ways which is proteinase-K (PK) digestion in normal condition A (Biorad proprietary detergent and chaotrope concentrations, 0.04 mg mL^−1^ PK) and denaturing condition Aʹ (5% [w/v] *N*-lauroylsarcosine sodium salt, 5% [w/v] sodium dodecyl sulphate, 0.11 mg mL^−1^ PK (Aʹ/A). Negatives do not show a PrP^res^ signal. The following normalised ratios Aʹ/A are indicative for CS, BSE and AS respectively > 1.43, between 0.78–1.43 and below 0.78.

### ISS-WB procedure for discrimination between classical scrapie and BSE (performed at ISS: Istituto Superiore di Sanità)

To discriminate between classical scrapie and BSE, the ISS discriminatory Western blot procedure (ISS-WB) was used. This test uses 0.2 mg mL^−1^ PK for digestion and antibodies P4 (0.4 µg IgG mL^−1^) and SAF84 (0.8 µg IgG mL^−1^) for detection. Data were collected with a chemo luminescence imager (VersaDoc, Bio-Rad) and quantified as in TSE EU Reference Laboratory manual. Two decisive cut-off values for BSE are applied: in BSE samples the P4/SAF84 ratios should be < 0.5 and molecular mass of the non-glycosylated PrP^res^ band (N, see footnote 2 for the triplet terms N, M and D) based on SAF84 < 0.5 kDa compared to that of internal control (sample I11) [41, 42].

### IZSTO-WB procedure for detection of Nor98/atypical scrapie (AS) (performed at IZSTO: Istituto Zooprofilattico Sperimentale del Piemonte at Torino)

Digestion with proteinase K (PK) and Western blotting (WB) using a chemo luminescence imager (ChemiDoc, Biorad) for data collection were followed as described before, except that for AS cases PK was used at 0.02 instead of 0.04 mg mL^−1^ [43]. Antibodies used were 12B2, 9A2, Sha31 and SAF84 respectively at concentrations 0.2, 0.2, 0.1 and 1 µg IgG mL^−1^.

### Triplex-WB procedure (performed at WBVR: Wageningen BioVeterinary Research)

PK was used at 0.05 mg mL^−1^, and WB performed with a mix of antibodies 12B2, Sha31 and SAF84 on a single membrane at concentrations of respectively 0.2, 0.1 and 0.5 µg mL^−1^ IgG. Calculations on resulting fluorescent antibody signals were carried out with ImageQuant software exactly as before [44]. The fluorescence of the mAbs with respective Zenon labels Alexa647, Alexa488 and Alexa555 (InVitrogen) was normalized to 1 based on recombinant ovPrP_ARQ_ on each gel. Molecular masses of PrP^res^ bands D, M and N (see footnote 2 for triplet band nomenclature) were estimated with Gel-Pro analyser software (Media Cybernetics) using as reference SeeBlue dye markers which are visible at 647 nm. Samples were analysed in triplicate. Parameters calculated from the Image Quant software figures such as molecular masses, 12B2/Sha31 ratios, M/D ratios, D-, M-, and N-fractions, and SAF84/Sha31 ratios at the 24 kDa PrP^res^ fraction yielded per sample standard deviations below respectively 4, 27 16, 5, 12, 23 and 17 in percentage of the average.

### Raising PK concentration to 1 mg mL^−1^ for PrP^res^ preparation (ISS-PK)

To investigate the PK susceptibility of CS cases, a new approach used a high concentration of PK (1 mg mL^−1^), or in some cases a range of PK concentrations between 0.02 and 4 mg mL^−1^ PK, followed by ISS-WB. After PK digestion at 1 mg mL^−1^, the P4/SAF84 ratio was calculated for each sample, relative to the ratio of an internal control (sample I11).

### Guanidine-treatment (ISS-Gdn)

To differentiate CS and CH1641-like isolates from small ruminant BSE a method to test structural stability of the PrP^Sc^ core was used [27]. The protocol (ISS-Gdn) included a pre-treatment with 3.5 M guanidine-HCl (Gdn). Equal aliquots of a sample were either left untreated or treated for 1 h at 37 °C and then adjusted to a final concentration of 0.35 M Gdn, and digested with 0.2 mg mL^−1^ PK. ISS-WB with PrP-core specific mAb SAF84 was used for detection. Stability of PrP^res^ core was reflected in the antibody binding signal ratios obtained at 3.5 and 0 M Gdn (3.5 M/0 M).

### High pH/PK treatment (WBVR-pH8)

Another method to test PrP^res^ core structural stability consisted of two digestion conditions where one aliquot of sample was digested at 50 µg mL^−1^ PK/pH 6.5 and another aliquot at 500 µg mL^−1^ PK/pH 8 [26]. WB was performed with PrP core specific antibody L42 (0.2 µg IgG mL^−1^) and chemo luminescence detection. Antibody signals on films were estimated as before [9]. The relative stability of the PrP^res^ core was expressed as the pH8/pH6.5 signal retention ratio between each set of aliquots.

### Statistical analyses

With statistical software (GraphPad Prism^®^ 8), one-way ANOVA compared three or more unmatched groups, based on the assumption that the populations were Gaussian. When *P* values were ≤ 0.05, means were considered to be derived from non-identical populations. In that case, one-way analysis of variance was used to establish whether differences between groups of data were greater (*P* ≤ 0.05) than expected using Bonferroni–Dunn t-test.

## Results

### Analyses to discriminate between BSE, classical scrapie and Nor98/atypical scrapie

**Figure 1:**
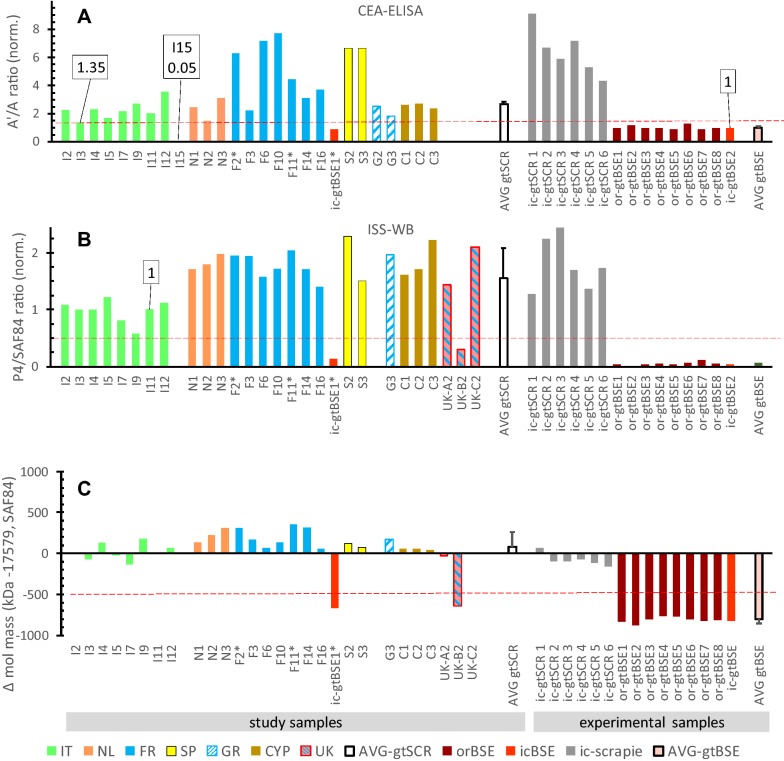
**Discriminatory test results for exclusion of BSE in goat samples.** Values below red broken lines indicate that sample concerned is considered BSE suspect. **A** CEA-ELISA. Values are normalised to sample ic-gtBSE2. **B**, **C** ISS-WB results (see Additional file 3). In **B** are shown *N*-*terminus* values relative to the PrP^res^ core as reflected in the P4/SAF84 ratios normalised to the ratio of sample I11. In **C** is shown the difference in molecular mass in kDa of the N-band between that in a sample and the reference sample (I11). AVG gtSCR and AVG gtBSE represent average and SD of respectively the classical scrapie field samples (except I15 and UKB2) and of all gtBSE samples. Ratios in **A** and **B** are inverted compared to original methodology for the logical reason that the bar heights correlate positivley with N terminus values of PrP^res^.

Initial analyses were carried out by CEA-ELISA on goat samples from all countries except on those from UK and G11–G17 from Greece. Most fields cases scored as CS with Aʹ/A ratios > 1.43, except for sample I3 which showed a borderline BSE value of 1.35 and sample I15 a ratio of 0.05 indicative for AS-like scrapie (Figure 1A). All experimental CS and BSE samples including ic-gtBSE1 resulted in values as expected for CS and BSE, respectively.

In ISS-WB analysis (see Additional file 3), most field cases fulfilled the two criteria for CS except for samples UK-B2 and I15 (Figures 1B and C). UK-B2 exhibited BSE-like features by showing both a low *N*-*terminal* epitope PrP^res^ content (P4/SAF84 signal ratio < 0.5) and N-band PrP^res^ molecular mass > 0.5 kDa lower than that of the CS reference I11.

The PrP^res^ banding pattern of sample I15 was as in AS-like samples with a major band at 8 kDa, when using antibody P4, while SAF84 did not show binding (see Additional file 3). This was further confirmed in IZSTO-WB with mAbs 12B2, 9A2, Sha31 and SAF84 (data not shown).

### Triplex-WB: three-antibody analysis on a single membrane

**Figure 2:**
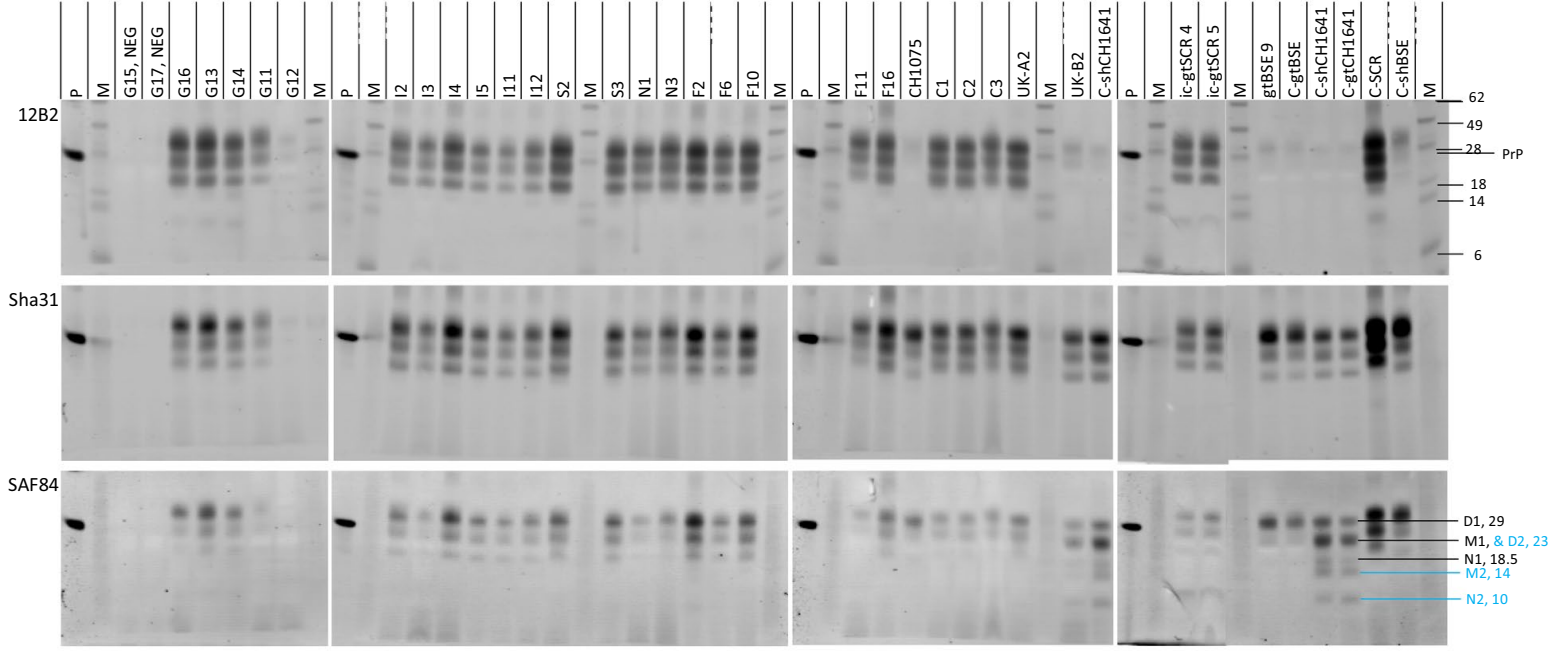
**Triplex-WB of goat study samples from different geographical regions together with TSE controls.** Three antibodies used are indicated left. Images are all taken from the same blotting membrane. Lanes P and M, respectively recombinant shPrP and molecular mass standards. Position of molecular mass standards are visible only in the 647 nm (12B2) image and are indicated with kDa figures. Sample identities as in Tables 1 and Additional files 1 and 2 are indicated above the lanes. Only the CH1641 controls and UK-B2 sample exhibit a unique glycoprofile difference between SAF84 and Sha31. In lane C-gtCH1641, the positions of the three bands in triplets PrP^res^#1 (black, D1, M1, N1) and PrP^res^#2 (blue, D2, M2, N2) are indicated in the SAF84 panel at the right, including their approximate molecular masses; M1 and D2 have nearly similar molecular masses. Therefore, the signal of SAF84 in the D2 + M1 area is clearly higher than in the D1 area because this co-migration reflects the sum of D2 and M1 with that antibody, while with antibodies having more *N*-*terminus* located epitope specificity than SAF84 such as Sha31 the D1-area is higher than the D2 + M1 region.” Tissue equivalents applied vary between 0.5 and 2 mg. Samples were analysed in triplicate WB experiments.

Triplex-WB can yield on one membrane quantifications of molecular properties of PrP^res^ from the signals of a mix of three mAbs which are in this study 12B2, Sha31 and SAF84 (Figure 2).

Similar results were obtained as above with ISS-WB but now they were estimated relative to the Sha31 signal instead to SAF84. The results can be summarized as follows:

1. in all but one case a high *N*-*terminal* epitope content with 12B2/Sha31 ratios between 0.3 and 1.2 were seen, the exception being UK-B2 (0.1, BSE-like) (Figure 3A); interestingly, the Italian samples as well as F16 and UK-A2 were the lowest in 12B2 epitope content (between 0.3 and 0.9) similar to what was observed with P4 in the ISS-WB (see Additional file 4). The *N*-*terminal* epitope content of this group of samples was in both WB systems statistically lower than of other CS samples and higher than of the BSE samples (*P* < 0.001).

**Figure 3:**
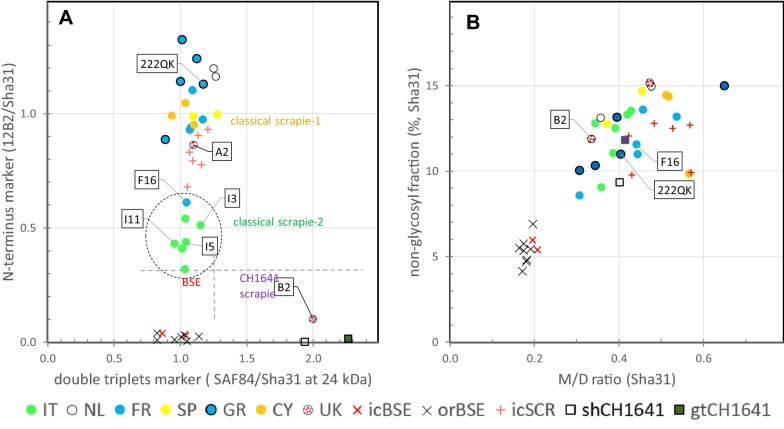
**Dot plots of data obtained from Triplex-WB of PrP**^**res**^ **as in Figure** **2**. Each dot represents an individual goat TSE sample analysed in triplicate. Symbols: circles represent field cases and colour the country of origin; other symbols represent experimental samples and control samples from sheep or goat. **A** Plot with marker for *N terminus* epitope level on vertical axis versus PrP^res^ double triplets marker on horizontal axis. Horizontal and vertical broken lines indicate the clear separation between CS, CH1641 and BSE. The lower *N*-*terminus* marker values of Italian cases and F16 are striking (encircled). **B** Triplet glycoprofile markers with non-glycosylated (N) fraction on vertical axis and on horizontal axis the ratio between signals in mono-glycosylated (M) and di-glycosylated fraction (D). A difference is obvious between BSE cases (low N and low M/D, due to high D levels in BSE samples) with CS and CH1641 cases.
2. a glycoprofile with M/D ratios > 0.3 can be observed in all study samples including UK-B2, gtCH1641 and shCH1641 due to a low D fraction in contrast to ratios ≤ 0.2 in BSE with high D levels. Additionally, the N band fraction was higher in all CS samples, including CH1641, than in BSE (Figure 3B).
3. molecular masses of the PrP^res^ N-fraction in the CS cases ranging from 19.3 to 21.6 kDa, and those of BSE, CH1641 and UK-B2 from 18.7 to 19.4 kDa. There was a reasonable linear regression correlation between *N*-*terminus* epitope content of (N + M + D bands) and the molecular mass of the N band (R^2^ = 0.602, see Additional file 5).

Banding patterns of Sha31 and SAF84 in UK-B2 were different from CS and BSE samples, but similar to that of control samples C-shCH1641 and C-gtCH1641 in which typically two PrP^res^ triplets were present (triplet #1 bands D1, M1 and N1, triplet #2 bands D2, M2, and N2 in Figure 2). Of these two triplets, one migrated between 18 and 29 kDa similar to that obtained with mAb Sha31 (PrP^res^#1), and the other between 10 and 24 kDa (PrP^res^#2) only bound by SAF84 (see Additional file 6). Proof for presence of such double triplet composition could be confirmed by using at 24–25 kDa signal of the SAF84 and Sha31 fractions in the 18–29 kDa region (ratio SAF84/Sha31 at 24 kDa), which in case of CH1641 yields a value around two while single populations are around one. All BSE and CS samples varied around one (range 0.8–1.2) (Figure 3A).

### PK sensitivity of PrP^res^*N*-*terminal* epitope of CS cases

**Figure 4:**
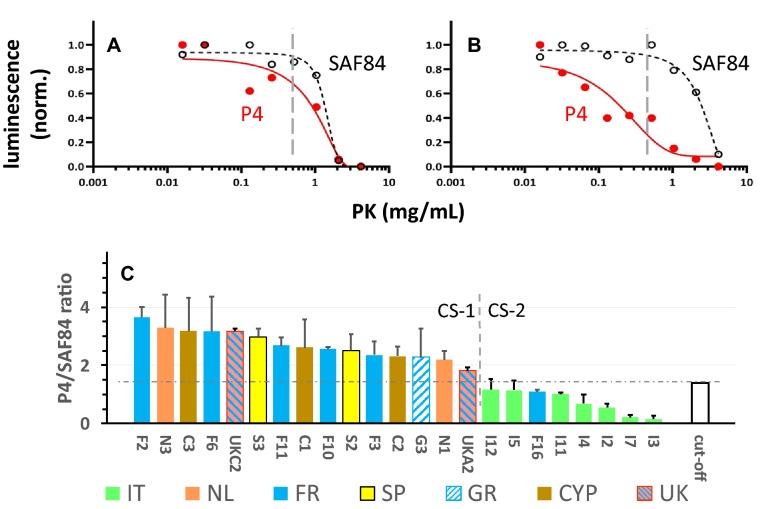
**Proteinase-K (PK) sensitivity of the** ***N*-*terminal*** **(P4) PrP**^**res**^ **epitope in goat classical scrapie (CS) samples. A**, **B** PK digestion curves of respective scrapie goat isolates S2 and I5, showing the differential sensitivities to PK of the epitopes of respectively SAF84 (black) and P4 (red). Vertical lines point to the 1 mg mL^−1^ PK condition used to study a larger selection of CS cases in **C**. **C** Shows the sensitivity of samples to PK at 1 mg mL^−1^ of the *N terminal* PrP^res^ P4-epitope relative to that of core epitope SAF84. Vertical dashed line separates the samples between the ones with highest (CS-1) and lowest (CS-2) P4 epitope content consistent with data in Additional file 4. The cut-off between the two groups is reflected by the horizontal dash line and is based on the value of the reference sample I11 plus the average value of the standard deviations of all samples (1 + 0.4 = 1.4).

The PK-sensitivity of the PrP^res^ *N*-*terminus* of CS cases in the two WB methods (see Additional file 4) was further tested by stepwise increasing the PK concentration from 0.02 to 4 mg PK mL^−1^ in several samples comparing the relative binding of P4 and SAF84 epitopes (Figures 4A and B). After confirming the reproducibility, all CS samples were subjected to one single PK digestion at 1 mg mL^−1^ to estimate the P4/SAF84 ratio (ISS-PK method). All Italian samples and F16 were clearly below a ratio cut-off value of 1.4 and considered as a separate group of CS type. These biochemical groups are here defined for > 1.4 and < 1.4 as type CS-1 and CS-2 respectively (Figure 4C).

### Structural stability of total PrP^res^

We also investigated the PK resistance of the PrP^res^ core region as an indicator of structural stability. This was carried out with two different approaches and WB to probe the effect.

**Figure 5:**
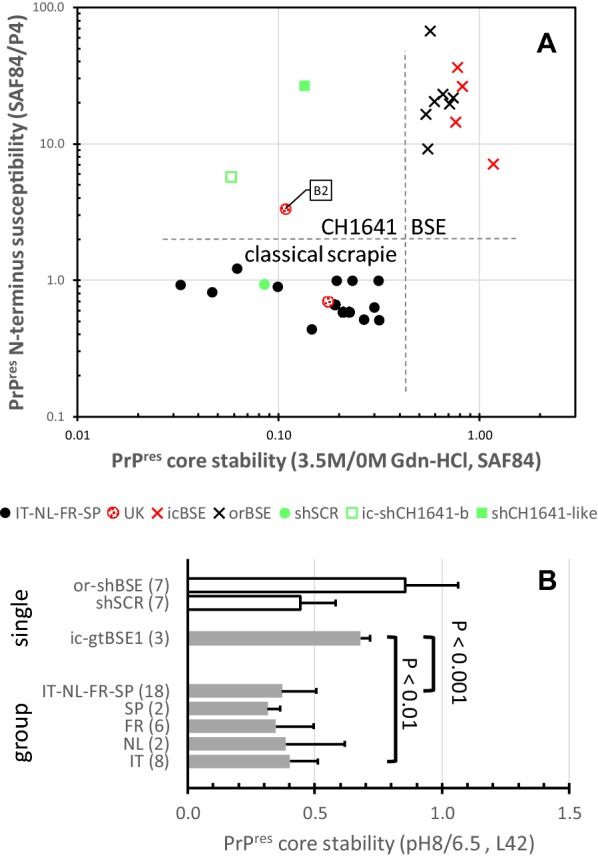
**Stability of PrP**^**res**^ **core region after denaturing and basic pretreatment of goat study samples. A** The effect of 3.5 M Gdn-HCl treatment on susceptibility to proteolytic degradation was compared to normal condition when analysed by ISS-WB with mAb SAF84. The denaturation yielded high core epitope losses in the CS and CH1641 samples, but not in BSE samples where the PrP^res^ core apparently is highly stable (horizontal axis). The vertical axis shows the susceptibility of the PrP^res^ *N*-*terminus* under normal conditions of the ISS-WB process. **B** The effect of basic pH8 pre-treatment on subsequent proteolytic degradation. Only samples from four countries were tested. The BSE samples appeared the most resistant against proteolytic degradation of the PrP^res^ core. All field samples from Italy, Netherlands, France and Spain together and separately from each country did exhibit a significantly higher susceptibility to Gdn denaturation than goatBSE, as indicated with the *P* values (Bonferroni–Dunn method).

After 3.5 M Gdn-HCl pre-treatment in the ISS-Gdn method, core epitope loss was probed by ISS-WB to measure the SAF84 signals at 3.5 M relative to that without pre-treatment. All CS study cases and CH1641 specimens were quite sensitive for PK digestion with 3.5 M/0 M ratios lower than 0.35 (i.e. > 65% core epitope loss) including UK-B2 (89% loss), while BSE samples were significantly more resistant with less than 45% loss (Figure 5A).

The WBVR-pH8 method compared high and normal pH during PK digestion. Expressed by the L42 signal ratio pH8/pH6.5, this yielded low and high retention ratios for CS and BSE, respectively (Figure 5B). While the CS cases on average lost around 62% (ratio < 0.38) of antibody binding, the goat BSE sample showed the lowest loss of binding up to only 36% (ratio > 0.64). A reference sample of sheep scrapie showed higher signal loss than sheep BSE—56% compared to 15%—in line with what had already been observed before [26].

## Discussion

The combined efforts in different laboratories, which shared the same goat brain macerates, enabled a thorough investigation using various chemical pre-treatments and subsequent biochemical analyses to clearly establish that none of the field cases was BSE.

On the other hand, the combined biochemical evidence from over 30 different field cases of prion disease collected from seven different European countries shows clearly that in goats similar types of scrapie occur as in sheep, which are atypical/Nor98 (AS) scrapie and several forms of classical scrapie (CS). Potentially three types of CS could be discriminated differing in increasing order of protease sensitivity of the N-terminus of PrP^Sc^: CS-1 occurring most frequently, CS-2 occurring—but probably not only—in Italy, and one unambiguous CH1641-like case found in a scrapie infected herd in the United Kingdom (see column Molecular TSE-type, Table 1).

### Biochemical parameters for typing TSEs

In this study, nine different molecular PrP^res^ parameters appeared useful to discriminate TSE types in brain homogenates of native goat samples (Table 2). These were glycoprofile (M/D and %N), PK resistance of the *N*-*terminus* (three approaches), molecular mass of PrP^res^ bands (reflected in the non-glycosylated fraction), double triplet composition, core sequence stability and—for AS—absence of a *C*-*terminal* fragment covering roughly the 154–234 PrP sequence corroborating a previous study [45].

**Table 2:** **PrP**^**res**^ **parameters that differentiate TSE-types in goats**

PrP^res^ parameter	CS-1	CS-2	CS-CH1641	BSE	AS
Glycoprofile^a^					
M%/D% ratio, Sha31	High (> 0.3)	High (> 0.3)	High (> 0.3)	Low (< 0.2)	NA
N%, Sha31	High (> 8%)	High (> 8%)	High (> 8%)	Low (< 8%)	NA
N-terminus level^b^					
Normal PK: 12B2/Sha31 ratio	High (0.8–1.3)	*Interm. (0.3–0.7)*	Low (< 0.2)	Low (< 0.2)	NA
High PK: P4/SAF84 ratio	High (1.4-4)	*Interm. (0.2–1.3)*	ND	ND	NA
High PK: A’/A ratio, SAF34 + Bar224	High (> 0.3)	High (> 0.3)	ND	Low (0.8–1.3)	minute (≪ 0.8)
kDa (N-band)^c^					
Triplex-WB, Sha31	19.9–21.6	*19.3–20.6*	18.5–19.5^e^	18.5–19.5	NA
ISS-WB, SAF84	17.5	17.5	16–17	16–17	8
Triplet profile, SAF84/Sha31 ratio at 22– 24 kDa	Single	Single	Double	Single	Absent
Core stability: SAF84 and L42	Low	Low	Low	High	NA
PrP C-terminus ~ 154–234^d^	Present	Present	Present	Present	Absent

Nine parameters of PrP^Sc^ obtained after differential PK digestion of TSE infected goat brain and WB. Epitope location determines the outcome. The italic texts indicate differences between CS-1 and CS-2. Between parentheses are the ranges in quantitative values as obtained in Triplex-WB (Sha31 related), ISS-WB (SAF84 related) or ELISA (SAF34 + Bar224 related). The ranges are relative to the different categories, not absolute, and should be compared per experiment with proper controls (BSE, CS-1, CS-2, CS-CH1641). ND: analysis not done, NA: not applicable due to absence of a classical PrP^res^ triplet. These typing tests may well work also on sheep TSE samples

^a^Glycoprofile estimated by ISS-WB with SAF84 did show similar discriminatory properties as with Sha31 for C-1, CS-2, and CH1641 M/D values > 0.4 and BSE < 0.4, and N percentages for CS > 13% and BSE < 13%

^b^Result with 12B2/Sha31 from Triplex-WB, P4/SAF84 from ISS-WB and SAF34 + Bar224 from CEA ELISA

^c^These two rows represent the same differentiating parameter. Differences between ISS-WB and Triplex-WB in molecular mass values are due to use of different gel systems and molecular mass standards

^d^Absence of PrP region ~ 154–234 is based on presence of Sha31 epitope and absence of SAF84 epitope corroborating interpretations by Pirisinu et al. [45]

^e^The molecular mass of the N-fraction of PrP^res^#1 triplet is estimated to be 18.5 kDa, and that of the PrP^res^#2 triplet 10 kDa (see Figure 2).

One of these parameters is a new candidate and dependent on a 1 mg mL^−1^ PK treatment that effectively leads to differentiation between the CS subclasses CS-1 and CS-2. While in the three tests using Western blotting (ISS-WB, Triplex-WB and ISS-P with high PK concentrations) the difference in PK susceptibility of the PrP^res^ *N*-*terminal* domain was obvious this was not the case in the ELISA. The explanation could well be that the ELISA is dependent on the presence of a more *N*-*terminally* located epitope between PrP amino acid residues 70–76 used in the ELISA compared to the P4 and 12B2 epitopes in these three WB tests which epitopes are located more down stream the PrP-sequence i.e. between residues 93–97. The ELISA is therefore more sensitive for removal of N-terminal amino acids at sites in the 70–93 amino acid region of PrP, which might be helpful in finding the deviant cases but not to recognize truly BSE-like cases.

For differentiating CS-CH1641 from BSE several robust parameters were available which are the two glycoprofile markers M/D ratio and percentage of the N-fraction, structural stability and the unique presence of two PrP^res^ triplets.

### Are biochemically distinct classical scrapie types related to different strains?

The different PrP^res^ signatures of the CS-1, CS-2 and CS-CH1641 cases might have at least two different origins. One would be that it is a host dependent phenomenon in which a common scrapie strain in certain hosts shows up with a PrP^res^ triplet property as observed under the current biochemical treatments for diagnosis. In this case, the host is determining the biochemical phenotype of the strain by yet unknown factors. The other possibility could be that the phenomenon is a real strain property, which in the particular in case of CS-CH1641 is even rarely observed in sheep and goats. If so, it should be possible to make scrapie strain types visible in transgenic mice with various ovine (or caprine) *PRNP* expression levels [46]. Also, the effect of PrP polymorphisms need to be considered. To figure this out quite a number of rodent models are nowadays available to enable such typing studies.

### Significance of TSE-type for resistance breeding and polymorphisms

As with sheep, rapid typing of potential TSE agents in goats is necessary since different types can have different genetic susceptibilities [47, 48] or even different zoonotic potential [49]. Resistance/susceptibility to TSEs in mammals including the human species is dependent on genetic variation in the *PRNP* gene coding sequence [50–53]. In goats this polymorphism variability is partly similar to that in sheep and currently at least 51 coding polymorphisms have been described in goat [54]. In our set of field cases goats with several *PRNP* genotypes were selected (Table 1), including two scrapie positive goats (G11 and G12) carrying a scrapie resistance related lysine at codon 222 in heterozygosity both of which contained very low PrP^res^ levels (see Additional file 2). However, there appeared to be no association between the variability in biochemical characteristics of PrP^res^ and *PRNP* genotype in this study. Breed of animals could be another reason for phenotypical variability but although the breed of most animals was known it is not possible to connect this information to our results by lack of sufficient samples and because within the breed itself PrP polymorphism distribution can greatly differ [55, 56].

### Geographical differences

Little is actually known about geographical differences with respect to the occurrence of prion strains. In this study on goats from seven European countries—Italy, France, Greece, Cyprus, Spain, Netherlands and United Kingdom—material was collected and distributed to participating partners from single macerates. From our stability experiments, PK treatments and the two different antibody combinations (P4/SAF84 and 12B2/Sha31/SAF84) used in the WB analyses, CS-2 is an example of geographic variation of scrapie types. This form does occur mainly both in mainland Italy and Sicily, and possibly also sometimes in other countries such as France (example F16). Whether this CS-2 type has a source in Italy in the use of a vaccine against *Mycoplasma agalactiae* in both goats and sheep during the late 1990s is a possibility [57]. CS-1 might have existed before in Italy, but maybe the vaccination strain has become the dominant one.

## Prospects

Similarities between sheep and goats in genetics and the prion protein sequence itself were also encountered in the TSE types discerned in this study on goat scrapie field cases. Our consortium will report separately whether these biochemical typing studies in the macerates are linked to any strain type after first passage in an unprecedented broad set of rodent models. So far it seems, that the CS-2 cases also in the rodent models point to a separate strain that underscores the importance of further developing biochemical tools for TSE type discrimination [58].

## Supplementary information

**Additional file 1. Experimentally generated goat scrapie and BSE isolates, and some control samples used in this study.** Table containing details of the reference and control samples used in the study.

**Additional file 2. Overview of the application of seven different biochemical analyses in goat and control samples.** Table containing goat sample sets used in the specific type of analyses performed.

**Additional file 3. ISS-WB of goat TSE brain samples from different geographical regions with antibodies P4 and SAF84.** Figure of ISS-WB with mAbs P4 and SAF84 on the set of goat study samples.

**Additional file 4. Sample ranking to their relative levels of** ***N*-*terminal*** **PrP**^**res**^ **epitopes of antibodies P4 and 12B2.** Figure in histogram form to compare the ranking of goat study sample series from high to low *N*-*terminal* epitope in ISS-WB and Triplex-WB.

**Additional file 5. Correlation between** ***N*-*terminus*** **data and molecular mass of the PrP**^**res**^ **non-glycosylated band obtained by Triplex-WB.** Figure in dotplot form showing the correspondence between total PrP^res^ *N*-*terminus* level and molecular mass of non-glycosylated PrP^res^ fraction.

**Additional file 6. Graphic comparison of the PrP**^**res**^ **double and single triplet state in resp. CH1641 and scrapie/BSE.** Figure showing difference in migration of PrP^res^ bands in CH1641 scrapie samples, goat study sample UK-B2 and reference TSEs as obtained with mAbs Sha31 and SAF84 in Triplex-WB.

## Footnotes

1 “TSE type” is used for phenotypic observations in infected tissues based on microscopic or test tube experiments. “Strain type” is used as the outcome from passaging of infected tissue in another host, usually rodents such as inbred mouse or bank vole lines, or transgenic mice expressing PrP from another species.

2 In biochemical terms, classical TSEs yields in Western blot analyses a triplet of PrP^res^ bands consisting of a di-, mono-, and non-glycosylated (resp. D, M and N) PrP fragment of similar amino acid sequence. For clarity in the use of uppercase N: non-glycosylated will be written with a regular capital N and amino terminus with the italics description *N-terminus*.
