# Supplementary material for: Four types of scrapie in goats differentiated from each other and bovine spongiform encephalopathy by biochemical methods
Source: Vet Res. 2019 Nov 25;50:97. doi: 10.1186/s13567-019-0718-z (PMC6878695; doi:10.1186/s13567-019-0718-z)
Supplement: Supplementary file 1 — Additional file 1. Experimentally generated goat scrapie and BSE isolates, and some control samples used in this study. Table containing details of the reference and control samples used in the study. [file 13567_2019_718_MOESM1_ESM.docx]

**Additional file 1 Experimentally generated goat scrapie and BSE isolates, and some control samples used in this study^a^.**

| study code | report | identity code | breed | genotype |
| --- | --- | --- | --- | --- |
| REPORTED EXPERIMENTAL CHALLENGE CASES | | | | |
| ic-gtSCR1 | Lacroux '14 | CP060075 | alpine-saanen/ENVT | 211RQ |
| ic-gtSCR2 |  | CP060247 | alpine-saanen/ENVT | 211RQ |
| ic-gtSCR3 |  | CP060087 | alpine-saanen/ENVT | 142IM |
| ic-gtSCR4 |  | CP060279 | alpine-saanen/ENVT | 142IM |
| ic-gtSCR5 |  | CP070151 | alpine-saanen/ENVT | wt |
| ic-gtSCR6 |  | CP070157 | alpine-saanen/ENVT | wt |
| or-gtBSE1 | Fast '17 | ZG34 | alpine-saanen/ENVT | wt |
| or-gtBSE2 |  | ZG38 | alpine-saanen/ENVT | wt |
| or-gtBSE3 |  | ZG20, | alpine-saanen/ENVT | 211RQ |
| or-gtBSE4 |  | 82x54 | boer-toggenburg/INRA | 211RQ |
| or-gtBSE5 |  | CH80171 | alpine-saanen/INRA | 211RQ |
| or-gtBSE6 |  | CH80316 | alpine-saanen/INRA | wt |
| or-gtBSE7 |  | CH80275 | alpine-saanen/INRA | wt |
| or-gtBSE8 |  | CH80582 | alpine-saanen/INRA | 211RQ |
| ic-gtBSE2 |  | CH0064 | alpine-saanen/INRA | wt |
| additional CONTROLS | | | | |
| C, shSCR1 | IZSTO | IT 03/18 |  | wt |
| C, shSCR2 | WBVR | NL 2003-34 |  | wt |
| C, gtNor98 | IZSTO | IT 04/17 |  | 154RH |
| C, boBSE | IZSTO | IT 02/18 |  | wt |
| C, ic-gtBSE3* | WBVR | X10008019-001 |  | wt |
| C, ic-shBSE* | WBVR | 6019555-8163 |  | wt |
| C, ic-gtCH1641* | WBVR | 8018337-1587 HS |  | wt |
| C, ic-shCH1641-a* | WBVR | NPU pool |  | wt |
| C, ic-shCH1641-b* | Roslin | 241/74 |  | 154HH |
| C, shCH1641-like | ANSES | FR 99–454 |  | 136VV |

^a^ The samples in this table originated from other published studies. The scrapie samples ic‑gtSCR ##1-6 were from intracerebral challenge study second passage where at first passage F10 was used [40]. The gtBSE samples ## 1-9 were collected in the oral challenge study in goats [39]; 1-4 were from 2^nd^ passage, ## 5-9 from 1^st^ passage. Control samples: origin of field cases are indicated with country initial together with identity code, experimental cases are marked with an asterisk. Samples C, ic-shCH1641-b and C‑shCJH1641-like are published in [27]. The specific tests performed on the samples as well as - when analysed in Triplex-WB - the PrP^res^ content of each sample are shown in Additional file 2.
