# Supplementary material for: Four types of scrapie in goats differentiated from each other and bovine spongiform encephalopathy by biochemical methods
Source: Vet Res. 2019 Nov 25;50:97. doi: 10.1186/s13567-019-0718-z (PMC6878695; doi:10.1186/s13567-019-0718-z)
Supplement: Supplementary file 2 — Additional file 2. Overview of the application of seven different biochemical analyses in goat and control samples. Table containing goat sample sets used in the specific type of analyses performed. [file 13567_2019_718_MOESM2_ESM.docx]

**Additional file 2: Overview of the application of seven different biochemical analyses in goat and control samples^a^.**

^a^ In top row after study code are per column shown the analysis concerned as detailed in the Materials and methods section. Details of the samples can be found in Table 1 and Additional file 1. ND, not determined.

^b^ Amount of PrP in ng mg^-1^ tissue equivalents (Teq) per sample. This was calculated on PrP^res^ in the samples by Triplex‑WB using as reference the recombinant sheep PrP_ARQ_ applied on each gel; < indicates that in the analysis the signal of the TSE sample remained in background.
