# Supplementary material for: Four types of scrapie in goats differentiated from each other and bovine spongiform encephalopathy by biochemical methods
Source: Vet Res. 2019 Nov 25;50:97. doi: 10.1186/s13567-019-0718-z (PMC6878695; doi:10.1186/s13567-019-0718-z)
Supplement: Supplementary file 3 — Additional file 3. ISS-WB of goat TSE brain samples from different geographical regions with antibodies P4 and SAF84. Figure of ISS-WB with mAbs P4 and SAF84 on the set of goat study samples. [file 13567_2019_718_MOESM3_ESM.docx]

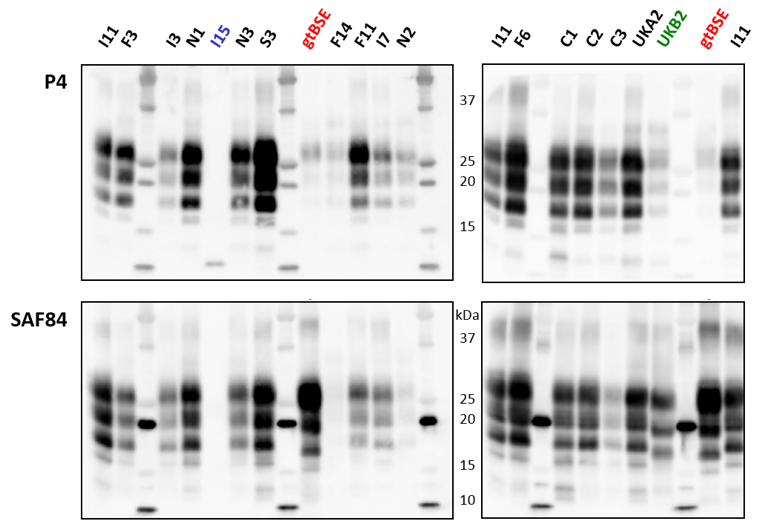


ISS-WB of goat TSE brain samples from different geographical regions with antibodies P4 and SAF84. Two separate SDS-PAGE gels were run and blotted in parallel and immuno‑stained with respective antibodies SAF84 and P4. Migration position of the molecular mass standards is indicated with their kDa. Only ic-gtBSE1 and UK-B2 did have highly reduced P4 epitope content. In the lane of the atypical/Nor98 case I15 only one band is visible with antibody P4 at 8 kDa while SAF84 is not showing any binding.
