# Supplementary material for: Four types of scrapie in goats differentiated from each other and bovine spongiform encephalopathy by biochemical methods
Source: Vet Res. 2019 Nov 25;50:97. doi: 10.1186/s13567-019-0718-z (PMC6878695; doi:10.1186/s13567-019-0718-z)
Supplement: Supplementary file 4 — Additional file 4. Sample ranking to their relative levels of N-terminal PrPres epitopes of antibodies P4 and 12B2. Figure in histogram form to compare the ranking of goat study sample series from high to low N-terminal epitope in ISS-WB and Triplex-WB. [file 13567_2019_718_MOESM4_ESM.docx]

Sample ranking to their relative levels of N-terminal PrP^res^ epitopes of antibodies P4 and 12B2. Panel A, P4 epitope relative to core epitope of SAF84 in ISS-WB. Panel B, 12B2 epitope relative to core epitope of Sha31 in Triplex-WB. As in Figure 1C, the P4/SAF84 ratios have been inverted for a similar representation of the N-terminus levels with 12B2/Sha31 ratios. The Italian cases exhibited in both WB systems the lowest N‑terminus epitope values. In addition, F16 and UKA2 have a similar position in both rankings. Values below the red dashed line indicate a BSE-like diagnosis. Western blots, from which panels A and B are derived, are presented respectively in Additional file 3 and Figure 2. Standard deviation (SD) per sample is presented by a vertical line in cases where a triplicate analysis was possible.
