# Supplementary material for: Four types of scrapie in goats differentiated from each other and bovine spongiform encephalopathy by biochemical methods
Source: Vet Res. 2019 Nov 25;50:97. doi: 10.1186/s13567-019-0718-z (PMC6878695; doi:10.1186/s13567-019-0718-z)
Supplement: Supplementary file 5 — Additional file 5. Correlation between N-terminus data and molecular mass of the PrPres non-glycosylated band obtained by Triplex-WB. Figure in dotplot form showing the correspondence between total PrPres N-terminus level and molecular mass of non-glycosylated PrPres fraction. [file 13567_2019_718_MOESM5_ESM.docx]

Correlation between N-terminus data and molecular mass of the PrP^res^ non-glycosylated band obtained by Triplex-WB. The round symbols represent the study cases with different colours per country of origin. There is a good correlation between both parameters in the 19.2-20.5 kDa range, with some exceptions e.g. I11 and I12. Linear regression line in the 18-22 kDa region estimated for the study cases including UK-B2 and ic‑gtBSE1 follows the mathematical equation y = 0.3442x, with R² = 0.602 (point 0,0 = at 18 kDa, 12B2/Sha31).
