# Supplementary material for: Four types of scrapie in goats differentiated from each other and bovine spongiform encephalopathy by biochemical methods
Source: Vet Res. 2019 Nov 25;50:97. doi: 10.1186/s13567-019-0718-z (PMC6878695; doi:10.1186/s13567-019-0718-z)
Supplement: Supplementary file 6 — Additional file 6. Graphic comparison of the PrPres double and single triplet state in resp. CH1641 and scrapie/BSE. Figure showing difference in migration of PrPres bands in CH1641 scrapie samples, goat study sample UK-B2 and reference TSEs as obtained with mAbs Sha31 and SAF84 in Triplex-WB. [file 13567_2019_718_MOESM6_ESM.docx]

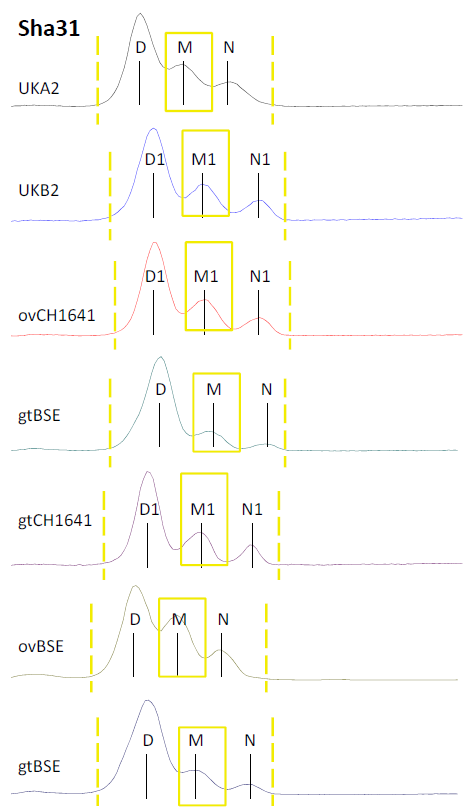

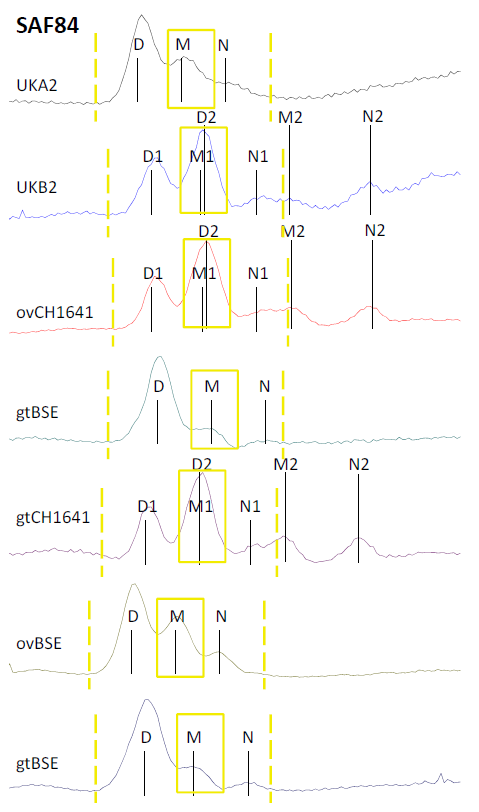


Graphic comparison of the PrP^res^ double and single triplet state in resp. CH1641 and scrapie/BSE. See for original Triplex WB picture Figure 2. Application of two PrP‑core specific antibodies in WB ‑ in this case Sha31 and SAF84 - unveils the unique PrP^res^ triplet property of CH1641 scrapie samples, and of the similarity of sample UK‑B2 with CH1641 scrapie. D, M, and N point to the migration position of respectively di‑glycosylated, mono­glycosylated and non‑glycosylated PrP bands in a PrP^res^ triplet. Where two populations are present, the D, M and N positions of PrP^res^#1 and PrP^res^#2 are labelled with respectively 1 and 2. The signal fraction (yellow blocks) within the triplet #1 region (yellow lines) is used to calculate the SAF84/Sha31 fractional ratio at the “24 kDa” region within the area surround with two dashed yellow lines.
